# Retinopathy Associated with Biallelic Mutations in *PYGM* (McArdle Disease)

**DOI:** 10.1016/j.ophtha.2018.09.013

**Published:** 2019-02

**Authors:** Omar A. Mahroo, Kamron N. Khan, Genevieve Wright, Zoe Ockrim, Renata S. Scalco, Anthony G. Robson, Adnan Tufail, Michel Michaelides, Ros Quinlivan, Andrew R. Webster

**Affiliations:** 1 UCL Institute of Ophthalmology, University College London, London, UK; 2 Genetics and Medical Retina Services, Moorfields Eye Hospital, London, UK; 3 Department of Ophthalmology, King’s College London, St Thomas’ Hospital Campus, London, UK; 4 Section of Ophthalmology and Neuroscience, Leeds Institute of Biomedical and Clinical Sciences, University of Leeds, Leeds, UK; 5 MRC Centre for Neuromuscular Disease, National Hospital for Neurology and Neurosurgery, London, UK

Identification of ocular associations with systemic diseases can aid diagnosis and phenotyping and can yield pathophysiologic insights. McArdle disease (glycogen storage disease type V) is a rare metabolic myopathy (estimated prevalence, 1/100 000) resulting from biallelic mutations in the *PYGM* gene, encoding muscle glycogen phosphorylase (reviewed by Lucia et al^1^). Patients experience exercise intolerance and risk acute rhabdomyolysis, although life expectancy is rarely affected. A case report from 1988 described pattern dystrophy of the retinal pigment epithelium (RPE) in an affected patient.^2^ Two further cases have been reported.^3^, ^4^ With only 3 cases, chance association is possible; the 2 entities may be unrelated.

Pattern dystrophies can be associated with mutations in a number of genes, most frequently *PRPH2*. We describe here a similar macular appearance in 4 further, unrelated patients with McArdle disease, including findings from multimodal retinal imaging and electrophysiologic examination. Genetic screening was performed, both to confirm *PYGM* mutations and to check for mutations in a number of known genes implicated in macular or pattern dystrophies. The latter screening showed negative results, suggesting that the 2 entities (retinopathy and McArdle disease) are indeed related. This study had research ethics committee approval.

Patient 1, a 64-year-old white man, was referred because of longstanding reduction in right eye vision and an unusual macular appearance. He reported exercise intolerance since childhood and had been diagnosed with McArdle disease after muscle biopsy. Unaided Snellen visual acuity was 20/80 in the right eye (20/60 with pinhole) and 20/20 in the left eye. Over the next 10 years, visual acuity deteriorated in the right eye to counting fingers but remained stable in the left eye. Patient 2, a 63-year-old South Asian man, was referred for reduction in left eye vision over the previous few years. McArdle disease had been diagnosed in late adulthood after muscle biopsy. Visual acuity was 20/17 in the right eye and 20/60 in the left eye; 4 years later, visual acuity was 20/20 and 20/80, respectively.

Patient 3, a 56-year-old white man, was noted previously to have an abnormal retinal appearance by his optometrist. He had been diagnosed with McArdle disease (molecularly confirmed) 2 years before review in our service. He was visually asymptomatic; visual acuity was 20/17 in each eye. Patient 4, a 68-year-old white man, was referred to his local hospital eye service after his optometrist noted an abnormal retinal appearance. He had no visual problems other than refractive error. He had been diagnosed by his local ophthalmology service, based on the macular appearance, with Stargardt disease. McArdle disease had been diagnosed after muscle biopsy in his 30s, although he reported fatigue since childhood. Visual acuity was 20/40 in the right eye and 20/30 in the left eye.

**Figure 1:**
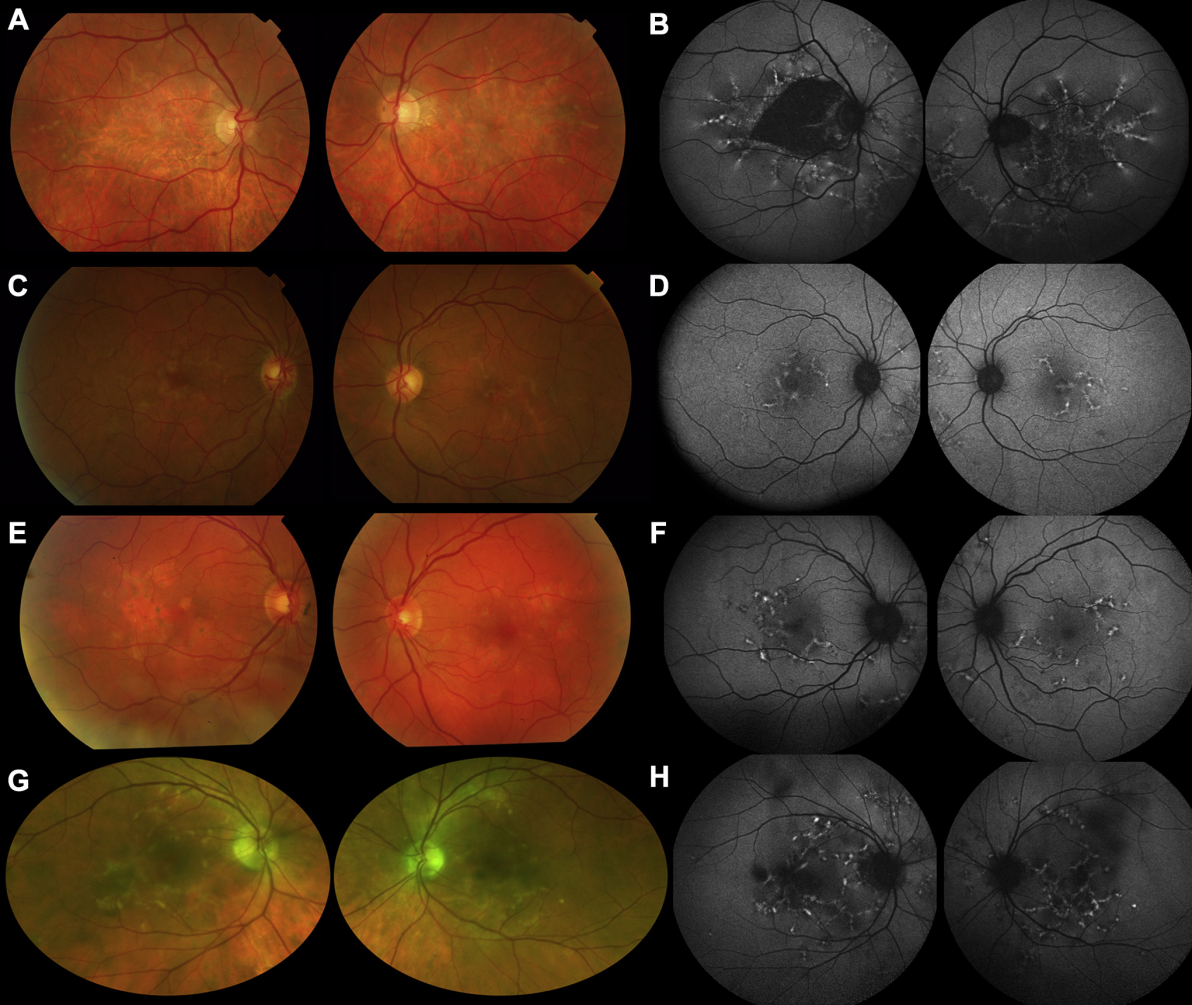
Retinal appearance on color imaging (left panels) and fundus autofluorescence (FAF) imaging (right panels). **A**, Color fundus images from patient 1. **B**, Short-wavelength (488-nm) posterior pole (55°) FAF images from patient 1. **C**, **D**, Corresponding images from patient 2. **E**, **F**, Corresponding images from patient 3. **G**, **H**, Pseudocolor image and 488-nm FAF image from patient 4. In all cases, yellow reticular lesions are visible on color imaging, with atrophy in patient 1 and small areas of pigmentary change in patients 2, 3, and 4. Fundus autofluorescence (FAF) shows reticular hyperautofluorescence (likely to result from loss of photoreceptor outer segments, accumulation of fluorophore in the retinal pigment epithelium [RPE], or both) in all cases, with additional dark areas indicating RPE atrophy in patient 1.

Figure 1 depicts the appearance of the fundus on color photography and autofluorescence, the latter showing distinctive reticular areas of stippled hyperautofluorescence. Spectral-domain OCT (Fig S1, available at www.aaojournal.org) confirmed abnormalities at the level of the RPE, outer retina, or both. Ultra-widefield autofluorescence imaging showed abnormalities in the far peripheral retina also, particularly nasally (Fig S2, available at www.aaojournal.org).

Patients 1, 2, and 4 underwent electroretinography recording according to international standards. All demonstrated normal full-field electroretinography results; pattern electroretinograms were undetectable in patient 1 and within normal limits in patients 2 and 4. Patients 1 and 2 also underwent electro-oculography, which showed a normal light rise.

Patients 1 and 2 underwent Sanger sequencing of all coding exons and exon-intron boundaries of *PRPH2*, thought to be the most likely genetic cause of such a fundus appearance (Manchester Centre for Genomic Medicine, Manchester, United Kingdom). No pathogenic variants were found. All patients underwent screening for disease-causing mutations in a number of additional genes implicated in macular or pattern dystrophies: *ABCA4*, *BEST1*, *CDH3*, *EFEMP1*, *ELOVL4*, *IMPG1*, *IMPG2*, *PROM1*, *PRPH2*, *TIMP3*; Molecular Vision Laboratory, Hillsboro, Oregon). For patients 2, 3, and 4, the panel also included *DRAM2*, *RP1L1*, and *TTLL5.* Results were negative.

Screening of *PYGM* (Sheffield Diagnostic Genetics Service, Sheffield, United Kingdom) molecularly confirmed McArdle disease in all patients. Patient 1 was homozygous for the frequently reported nonsense mutation p.(Arg50*). Patient 2 harbored the novel homozygous stop mutation p.(Gln176*). Patients 3 and 4 were compound heterozygotes: patient 3, p.(Arg50*) and p.(Gly205Ser); patient 4, p.(Arg94Trp) and p.(Gly695Arg).

In all patients, diagnosis of McArdle disease preceded detection of retinal abnormalities (apart from patient 3). Patients 1 and 2 were referred to the ophthalmology service because of visual symptoms; patients 3 and 4 were visually asymptomatic, but an optometrist detected an abnormal fundal appearance. Genetic testing for macular or pattern dystrophy genes was initiated by our service, and genetic confirmation of McArdle disease also was obtained after review in our service (except in patient 3).

This article reports multimodal imaging findings of a distinctive retinopathy in 4 unrelated patients with McArdle disease, similar to the previous case reports. We also report results of retinal electrophysiologic analysis: despite far peripheral abnormalities on ultrawide-field imaging, there was no definite electrophysiologic evidence of generalized retinal, or generalized RPE, dysfunction (full-field electroretinography and electro-oculography light rise results were normal).

Screening results for mutations in a number of macular dystrophy genes were negative. In particular, the findings could not be attributed to mutations in *PRPH2*, which would have been most likely to result in a similar retinal phenotype. Patient 4 had been diagnosed previously with Stargardt disease, but genetic testing yielded no mutations in *ABCA4*. The results of this study are of clinical significance because they support the association of this retinopathy with McArdle disease and can reduce the likelihood of misdiagnosis; this is increasingly important because particular genetic causes of retinopathy (including *ABCA4*) are subject to a number of novel treatment trials.

Why might muscle glycogen phosphorylase deficiency affect the retina? The enzyme catalyses conversion of glycogen to glucose-1-phosphate in skeletal muscle. Liver, muscle, and brain have different isoforms of the enzyme. In human RPE, glycogen is present, and both muscle and brain isoenzymes have been demonstrated. Intracellular glycogen is likely to act as a buffer^5^ in glucose delivery from choroid to photoreceptors via the RPE. It is possible that deficiency of a glycogen phosphorylase isoenzyme may disturb this process, leading to degeneration. This is one hypothesis; other mechanisms are possible, and further work is needed.
