# Supplementary material for: Retinopathy Associated with Biallelic Mutations in PYGM (McArdle Disease)
Source: Ophthalmology. 2019 Feb;126(2):320–2. doi: 10.1016/j.ophtha.2018.09.013 (PMC6347563; doi:10.1016/j.ophtha.2018.09.013)
Supplement: Figure S1 [file mmc1.pdf]

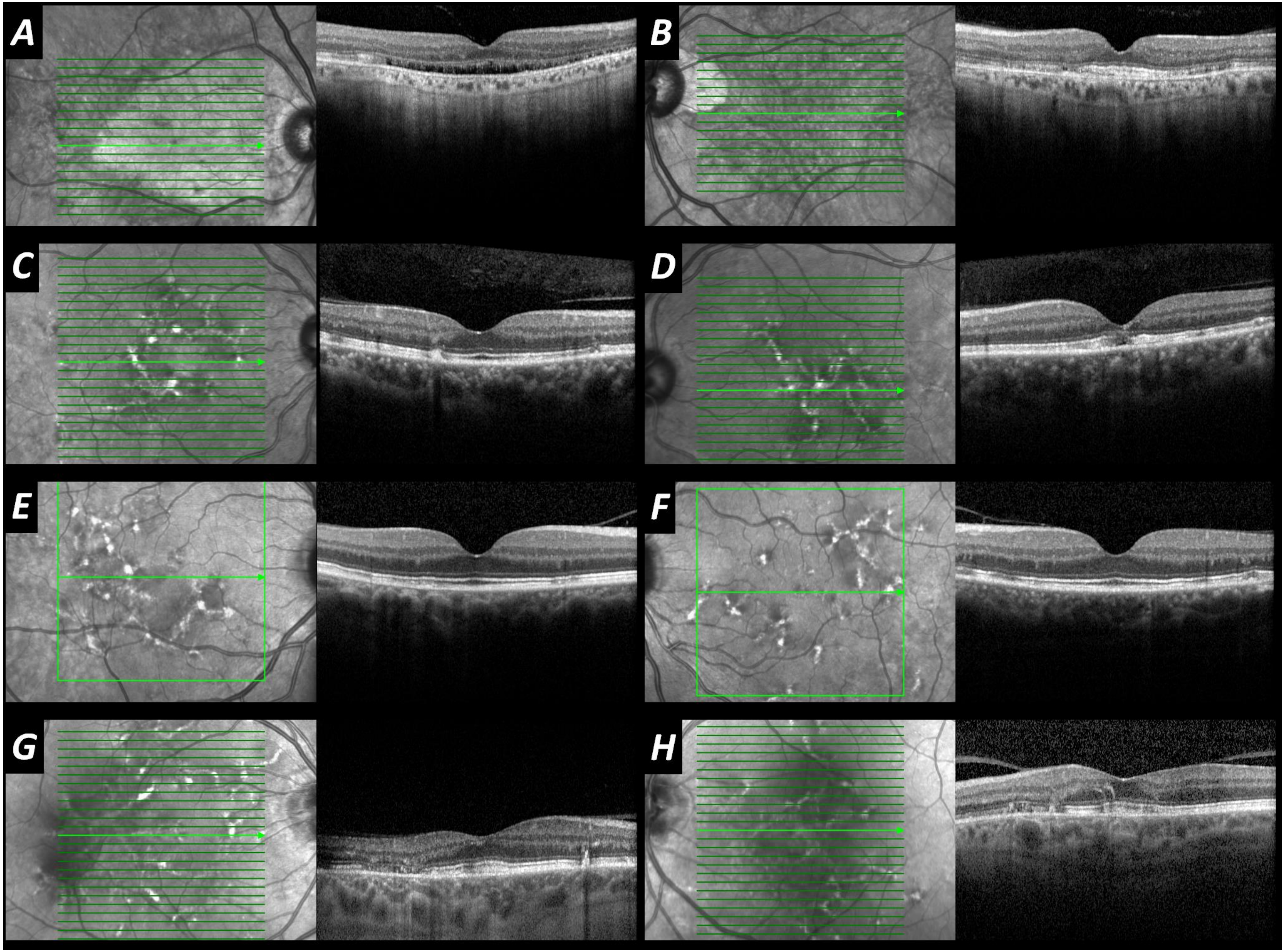

Supplementary Figure 1. Near infra red reflectance (NIRR) and spectral domain optical coherence tomography (OCT) images. In each panel, NIRR images are on the left, and OCT images (horizontal scan through foveal centre) are on the right. *A-H*, Images from Cases 1-4. OCT scans confirm that abnormalities are present in the RPE and outer retina. Focal areas of abnormal reflectivity are seen at the level of the photoreceptors and RPE, with hyperreflective deposits in some cases (appearing to correspond with hyperreflective areas on NIRR). The right eye of Case 1 (*C*) shows a hyporeflective space on OCT suggesting loss of RPE and overlying photoreceptor outer segments. OCT imaging of the left eye in Case 4 also shows two areas of hyper-reflectivity in the outer nuclear layer, which are likely to correspond to photoreceptor axons.
