# Supplementary material for: Retinopathy Associated with Biallelic Mutations in PYGM (McArdle Disease)
Source: Ophthalmology. 2019 Feb;126(2):320–2. doi: 10.1016/j.ophtha.2018.09.013 (PMC6347563; doi:10.1016/j.ophtha.2018.09.013)
Supplement: Figure S2 [file mmc2.pdf]

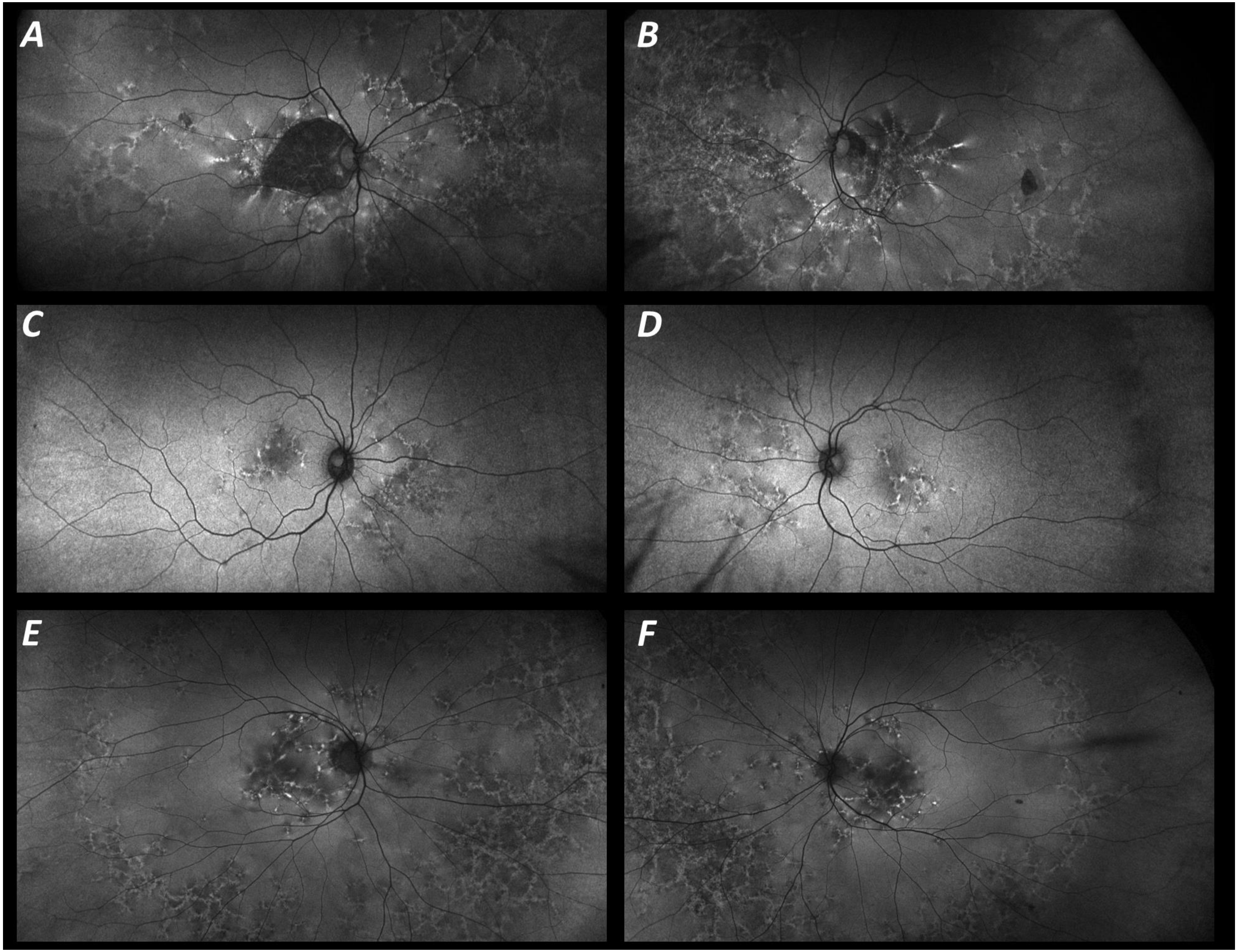

Supplementary Figure 2. Ultra-widefield autofluorescence (532 nm) images. Images are from Cases 1 (*A,B*), 2 (*C,D*) and 4 (*E,F*). *A,C,E,* right eyes; *B,D,F,* left eyes. The central areas show the same abnormalities as in Figure 2. The far peripheral areas also demonstrate abnormalities to varying degrees showing that the changes are not confined to the macula.
